# A neuronal signature of accurate imitative learning in wild-caught songbirds (swamp sparrows, *Melospiza georgiana*)

**DOI:** 10.1038/s41598-017-17401-2

**Published:** 2017-12-11

**Authors:** Dana L. Moseley, Narendra R. Joshi, Jonathan F. Prather, Jeffrey Podos, Luke Remage-Healey

**Affiliations:** 1 Department of Biology, University of Massachusetts, Amherst, MA 01003 USA; 2 Smithsonian Institution, Migratory Bird Center, National Zoological Park, PO Box 37012 MRC 5503, Washington, DC 20013-7012 USA; 3 Department of Biology, James Madison University, Harrisonburg, VA 22807 USA; 4 Department of Psychological and Brain Sciences, University of Massachusetts, Amherst, MA 01003 USA; 5 Department of Zoology and Physiology, Program in Neuroscience, University of Wyoming, Laramie, WY 82071 USA

## Abstract

In humans and other animals, behavioural variation in learning has been associated with variation in neural features like morphology and myelination. By contrast, it is essentially unknown whether cognitive performance scales with electrophysiological properties of individual neurons. Birdsong learning offers a rich system to investigate this topic as song acquisition is similar to human language learning. Here, we address the interface between behavioural learning and neurophysiology in a cohort of wild-caught, hand-reared songbirds (swamp sparrows, *Melospiza georgiana*). We report the discovery in the forebrain HVC of sensorimotor ‘bridge’ neurons that simultaneously and selectively represent two critical learning-related schemas: the bird’s own song, and the specific tutor model from which that song was copied. Furthermore, the prevalence and response properties of bridge neurons correlate with learning ability – males that copied tutor songs more accurately had more bridge neurons. Our results are consistent with the hypothesis that accurate imitative learning depends on a successful bridge, within single cortical neurons, between the representation of learning models and their sensorimotor copies. Whether such bridge neurons are a necessary mechanism for accurate learning or an outcome of learning accuracy is unknown at this stage, but can now be addressed in future developmental studies.

## Introduction

As with human speech, vocal learning in many songbirds involves two discrete processes: a sensory phase during which vocal models are memorized, and a sensorimotor phase during which memorized models are recalled and reproduced with increasing accuracy. Fundamental to the second phase is an interplay between neural representations of the original model and the emergent vocalizations produced by the learner^1^. The neural substrates of these two learning-related schemas (tutor model and learner copy) are essentially unknown, and identifying them would ideally involve longitudinal studies in which early learning and subsequent vocal practice are chronologically separated.

Swamp sparrows (*Melospiza georgiana*) are a well-suited species for studying vocal learning. Young male swamp sparrows memorize a variety of tutor songs, and each male learns to sing a repertoire of multiple song types, each of which can be readily quantified and usually are attributable to a particular tutor song. An important feature of swamp sparrow song is that syllable type is synonymous with song type, as swamp sparrows sing a learned, repeated trill composed of a single syllable. In this species, the sensory and sensorimotor phases are separated by many months, providing a natural means to disambiguate the neural basis of sensory and sensorimotor learning. As with other songbirds^2^, song learning in juvenile swamp sparrows is mediated by a network of discrete, interconnected brain regions that span the sensorimotor cortex, premotor cortex, and striatum^3–7^ (note: the songbird pallium is considered anologous to mammalian cortex^6,8–10^). This network is essential for song learning and performance, but there are substantial differences between individuals in the accuracy of their song learning^1^. Together, these features enable exploration of the neural basis of individual variability in learning outcomes.

Here we wild-caught and hand-reared 13 male swamp sparrow nestlings and exposed them to a common set of ten tutor songs, comprised of two control song types at wild-recorded trill rates and eight songs with trill rates increased to varying degrees to challenge birds’ vocal performance capacities during learning. This training paradigm results in wide variation in individual learning competency^11^. Our study tested the hypothesis that variation in vocal-learning ability has a neurophysiological correlate that can be identified at the level of single neurons. Prior work has shown that single neurons in the HVC are selective for the bird’s own song (BOS)^12,13^, and that neurons can be dually selective^14^. Here we investigate how the selectivity of HVC neurons relates to song development and the learning accuracy reflected in adult crystallized song.

## Results and Discussion

### Song Learning

As adults, our subjects crystallized a total of 33 songs (repertoire sizes = 2.4 ± 0.24, mean ± SEM, song types per subject) with some males crystallizing the same song type as others. For crystallized song types that could be identified as copies of specific tutor songs, imitative accuracy varied widely and ranged from 44% to 78% structural similarity to the best-matched tutor song, as measured by pairwise spectrographic cross-correlation (SPCC) scores^15,16^. We examined SPCC both at the level of syllable and at the level of individual notes for each copy compared to the ten tutor songs. Four males reproduced complex syllables with accurate note types and sequence so that copies could be attributed to a specific tutor model by eye and by SPCC. Five other males appeared to copy a tutor song with errors, but the copy could be matched to the corresponding model using SPCC of both the syllable and individual notes comprising the syllable. Finally four males produced song types that lacked complex syllable structure and consisted of merely one note repeated in a trill, which thus could not be attributed a sole tutor model, but rather matched the similar note-type in multiple tutor songs.

To explore the neuronal underpinnings of imitative accuracy, we conducted *in vivo* recordings from sites within the sensorimotor nucleus HVC. Guided by established coordinates and the characteristic, vigorous multi-unit responsiveness to acoustic playback of bird’s own song (BOS; see Methods), we identified HVC in each subject, recorded neural activity in response to a playback regime, and discriminated single units.

### Single Unit Recordings

We isolated 86 single HVC units (n = 13 birds, mean ± SEM = 6.77 ± 1.50 single units per bird). As is typical of HVC in songbirds, 81 or the 86 total single units showed significant responses to one or both of the birds’ own songs (BOSs) (t-test, p < 0.05), and 49 of those 81 were also specifically selective for BOS versus novel conspecific songs (d’CON mean ± SEM = 0.92 ± 0.05; criteria as in^17^), and the rest did not meet criteria for selectivity. Consistent with prior work demonstrating that the swamp sparrow HVC also maintains persistent representations of tutor songs into adulthood^17^, 82 of the 86 HVC units showed significant responses to one or more tutor songs (t-test, p < 0.05).

Each of the ten tutor songs elicited significant auditory responses (t-test, p < 0.05) from a large portion of the population that we sampled (range: 22–31 cells responded to each tutor song per bird). Further, each tutor song evoked selective auditory responses from an average of 3.8 cells per bird sampled (range 1–14 cells, showed selectivity for a tutor vs. novel conspecific song, criterion d’CON > 0.5). The variation among individual males in the accuracy of their song copying was therefore not attributable to general under-representation in HVC of any particular tutor model, even for those models originally presented at higher performance trill rates. Despite constraints in both vocal motor reproduction (vocal performance^11,18^) and auditory representation of songs with accelerated trill rates (auditory phase-locking^19^), we could spectrographically attribute adult copies of songs to tutor models that had been accelerated up to 140% of their natural trill rate. There were no apparent differences between neuronal responses to accelerated tutor models versus models presented at the natural trill rate (2.7 units per bird were significantly responsive to high trill rate songs as compared to 3.0 units per bird for control songs). Therefore, males reared with high trill-rate tutor models were able to represent them in HVC as adults, and some males successfully reproduced these accelerated song models in their copies.

### Tutor Selectivity

A prominent outcome of our study was that for some HVC units, the response to a single tutor song greatly exceeded responses to all other tutor songs. To quantify this selectivity, we generated a “Tutor Selectivity Index” (TSI), modified from the psychophysical parameter *d’* (see^20,21^), which measures the selectivity for one stimulus over another using each stimulus’s response strength (RS) calculated by subtracting the prior spontaneous firing rate from the firing rate during the stimulus, as follows in Equation 1.

$$TSI=\frac{2(R{S}_{TUT1st}-R{S}_{TUT2nd})}{\sqrt{({{\sigma }^{2}}_{TUT1st}+{{\sigma }^{2}}_{TUT2nd})}}$$

Equation 1: Tutor Selectivity Index (TSI), where RS_TUT1st_ and RS_TUT2_ are response strengths (RS) of the HVC unit to tutor songs with the highest and second highest d’CON, respectively; and σ^2^ factors are the corresponding RS variances. As with *d’*, a higher positive value for TSI indicates a stronger selectivity for a single tutor song relative to all others.

We used this conservative TSI measure along with established criteria for auditory responsiveness and selectivity, such as d’ to novel (unfamiliar) conspecific songs, in order to determine inclusion of single units in tutor-selective categories (see Table 1).

**Table 1:** Criteria for unit categories.

	d’CON	TSI (Tut1)	t test	TSI(Tut1) as compared to TSI(BOS)
BOS Selective	>0.3	NA	p < 0.05	TSI(BOS) > 0.5*TSI(Tut1)
Broadly Tutor Selective	>0.3^#^	NA	p < 0.05^#^	NA
Sharp (Tutor Selective)	>0.3	>0.125	p < 0.05	TSI(Tut1) > 0.5*TSI(BOS)
Bridge (both Tutor & BOS selective)	>0.3	>0.3*	p < 0.05	0.5*TSI(Tut1) < TSI(BOS) < 2*TSI(Tut1)

All abbreviations are as in the main document. Units were required to achieve all criteria to be included as “sharp” or “bridge” cells in results. Because TSI is a conservative measure of selectivity over a conspecific tutor song, as opposed to a hetero-specific or reversed song, we used a lower cut-off than the established d’(HET or REV) > 0.5.

^#^Indicates that the t-tests were significant for three or more tutor stimuli and the d’CON was > 0.3 for three or more tutor stimuli for each cell in the broadly tutor selective cells.

*Bridge cells were determined from 44 cells that were considered for inclusion as bridge cells, the median of the TSI = 0.209, and therefore we set a cutoff for TSI at TSI > 0.3 and set other parameters for inclusion as described in the table.

Sharp cells were determined by a pronounced response to tutor song while exhibiting little to no response to BOS, and thus we set a cutoff for TSI lower than that of bridge cells at TSI (sharp) > 0.125.

**Figure 1:**
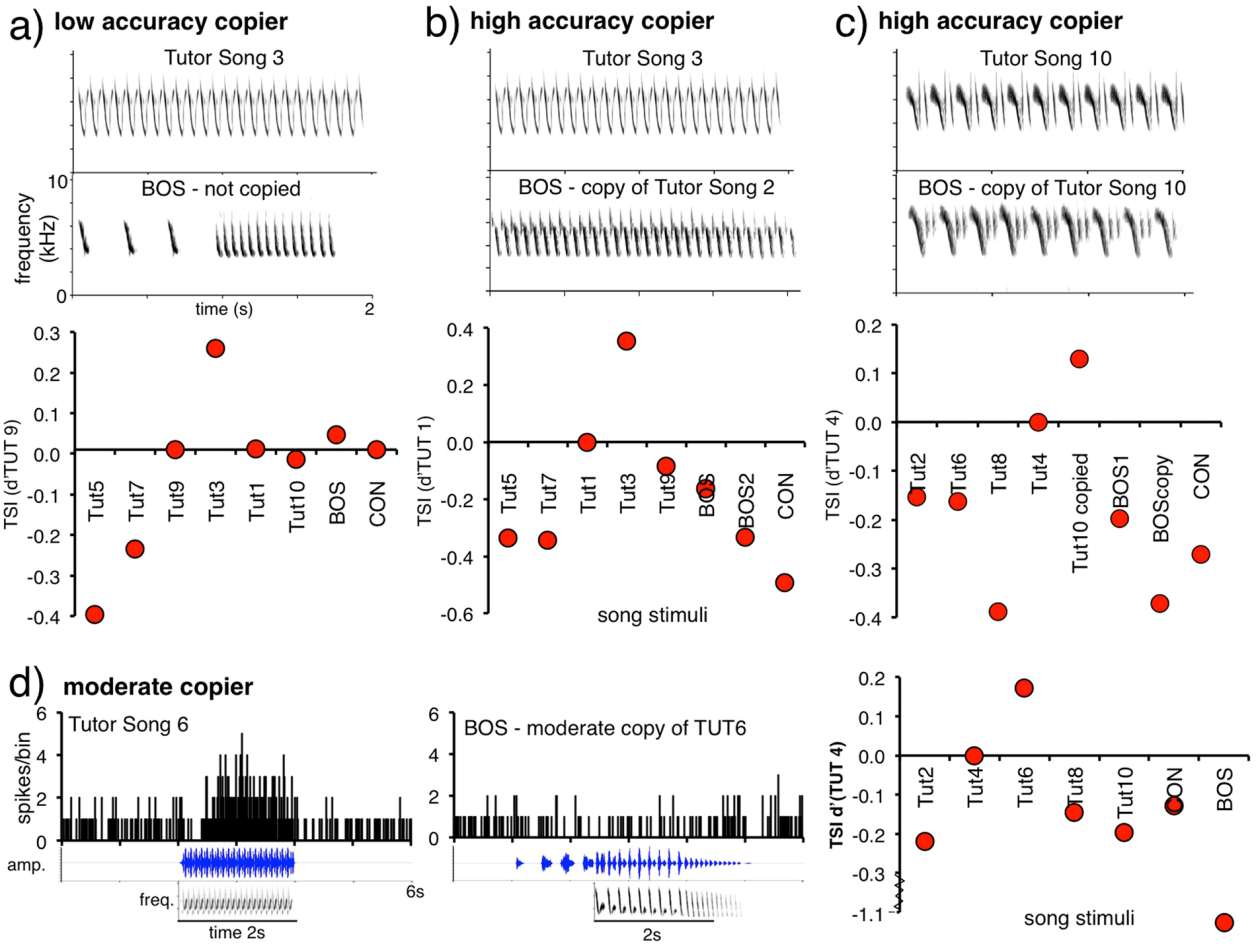
Swamp sparrows varied in their learning accuracy, but HVC sharp cells were all selective for a single tutor song over all other stimuli. (**a**–**c**) Example spectrograms from tutor and learner songs (top) and summary TSI values (bottom) for sharp cells from corresponding birds with varying learning outcomes. TSI measures selectivity to stimuli relative to the tutor song with the second-highest evoked response for each unit. All sharp cells were significantly responsive and highly selective to one tutor song. Sharp cells were found in birds with a variety of learning outcomes, e.g., males that did not sing an accurate copy of any tutor song but for whom HVC had sharp selectivity for a specific tutor song (**a**), males who sang an accurate copy for whom HVC had sharp selectivity for a different tutor song, not reproduced in their repertoire (**b**), and males who sang highly accurate copies and for whom HVC had sharp selectivity for the tutor song copied (**c**). (**d**) Peristimulus histograms (PSTH, spikes per bin) of a representative ‘sharp’ single unit to playback of the highest response-eliciting tutor song stimulus and to playback of BOS, waveforms and spectrograms below each and TSI values, right.

We identified 20 HVC units from our sample (23%) that had additional distinctive properties and fell into two categories of interest. First, some units were highly selective for a single tutor song over all other stimuli, including all BOS stimuli (Fig. 1; 8 units; TSI mean ± SEM = 0.253 ± 0.035; d’CON = 0.44 ± 0.07; Table 1). For these “sharp cells”, selectivity (TSI) for each unit’s top tutor song was at least twofold higher than that for any BOS in every case (d’BOS mean = 0.654 ± 0.152), indicating that each of these cells was selective for a specific tutor song alone. Moreover, the d’CON values for BOS in sharp cells were noticeably low (mean ± SEM = −0.19 ± 0.5). Thus, sharp cells appear to represent a key cognitive schema – tutor song – with high selectivity. Some sharp cells were selective for tutor songs that birds did not copy (Fig. 1a), replicating and confirming results reported previously^17^, yet others were selective for tutor songs that birds had copied with high fidelity, which had not been reported previously (e.g., Fig. 1c). This combination of outcomes suggests that the population of sharp cells in the adult HVC maintains a library of tutor song representations regardless of whether those tutor songs eventually provide models for copies emerging in adulthood.

**Figure 2:**
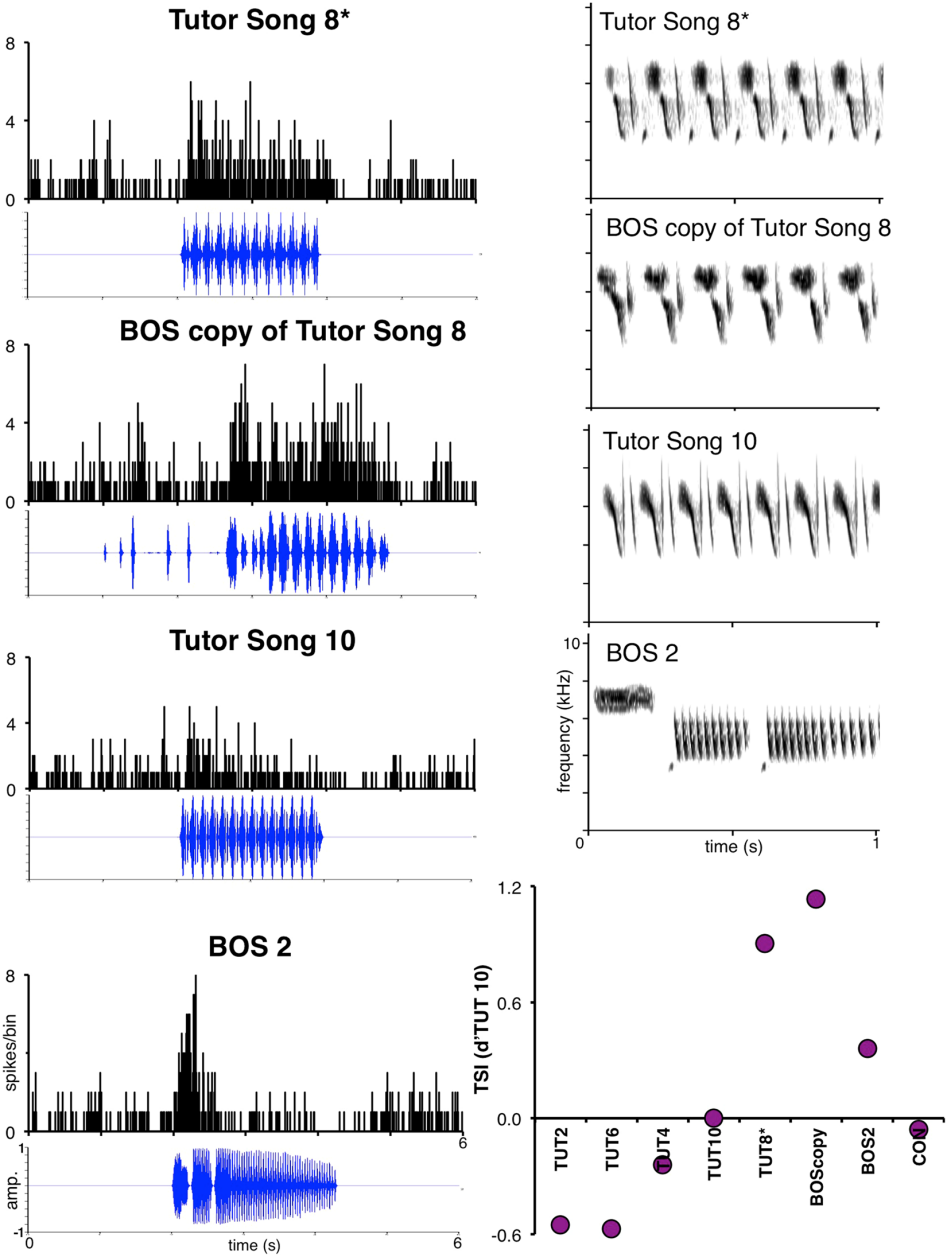
HVC bridge cells were selective for single tutor songs and birds’ own copies of those tutor songs. Left: PSTH for a representative bridge cell in response to the tutor song from which the male copied (as indicated by an asterisk, TUT8), the bird’s own copy of that tutor song (BOScopy SPCC at 74%), the tutor song that generated the second highest neural response as reference (TUT10, SPCC at 48%), and a second BOS (BOS2) from the bird’s repertoire as reference. Spectrograms shown for all these stimuli pictured at right. Though BOS2 showed some peak spiking, this spiking was precisely associated with introductory notes, and spiking did not persist at a similar magnitude throughout the trilled portion (the learned portion) of the song. TSI values for stimuli presented to this unit at bottom right.

A second category of neurons, which we discovered in the HVC of adult swamp sparrows, we termed “bridge cells.” Bridge cells were selective for two stimuli above all others presented – a single tutor song *and* the bird’s own copy of that tutor song (Fig. 2; 12 units; selectivity for the copied tutor song: TSI = 0.544 ± 0.099, d’CON = 0.593 ± 0.071; selectivity for the BOScopy: TSI = 0.794 ± 0.113, d’CON = 0.793 ± 0.121; Supplementary Table 1). For swamp sparrows that learn multiple song types from different tutor songs, the observation of a dual response for a specific combination of songs led us to evaluate the following ideas.

To investigate the possibility that bridge cells might respond to both the tutor song and the bird’s copy of that song merely because both stimuli share a similar acoustic structure, we quantified the acoustic properties of each of those songs. Birds with bridge cells varied widely in the degree of structural similarity between the selected tutor song and their copy of that song (mean = 0.61, range = 0.44–0.78). In fact, the degree of acoustic similarity between BOS and the corresponding tutor song did not predict the strength of auditory responses that those stimuli evoked in bridge cells (Supplementary Figs S1 and S2). In other words, songs that were better acoustic matches to tutor songs did not elicit stronger neural responses to tutor songs.

### Bridge cells and learning ability

**Figure 3:**
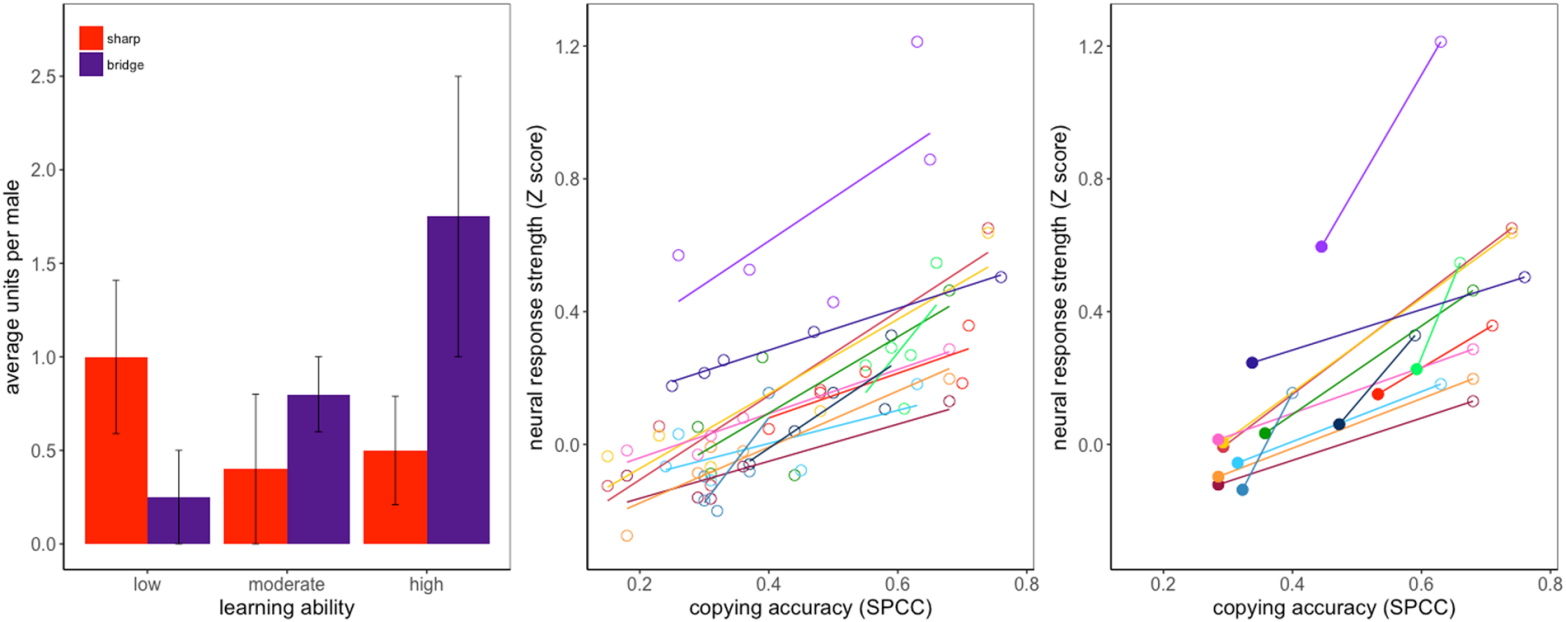
Sharp cells were found in birds regardless of learning ability, whereas bridge cells were more prevalent in birds that copied tutor songs with higher accuracy. Bridge cell response strength also strongly covaried with the match between the response-generating tutor song-model as compared to the match to the other tutor stimuli. (**a**) The average number of sharp cells per male (red) did not differ between learning ability group (n = 13, Mann Whitney U p > 0.05), whereas the average number of bridge cells (purple) varied across learning category with “high-accuracy” learners possessing significantly more bridge cells than “low-accuracy” learners (n = 13, Mann Whitney U_13,23_ p < 0.05). A similar number of single units were recorded from each group (28 low, 38 moderate, and 25 high). (**b**) For every bridge cell (n = 12), Z-score values for the response to each of the five presented tutor songs are regressed against the copying accuracy (SPCC value) of the BOS to those five tutor songs. Each color represents a cell, and each circle represents one of the five tutor stimuli played to that male. (**c**) The same data (as in **b**) summarized as two points per cell: the tutor song for which the cell was most selective (highest TSI, rank 1, open circles), and the mean of the four tutor songs for which the cell was not selective (TSI ranks 2–5, filled circles). Males possessing bridge cells showed a positive relationship between copying accuracy for the top tutor song and bridge cell neural response strength for tutor songs (all slopes were positive and differed significantly from zero F = 5.37, p < 0.0001).

In swamp sparrows and other songbirds, auditory and motor representations of BOS are thought to be enabled by HVC mirror neurons^22,23^. We therefore hypothesized that dual representation of model songs and their sensorimotor copies in bridge cells could be associated with the accuracy of imitative learning. As a first step in addressing this hypothesis, we asked whether the prevalence and response properties of HVC bridge cells corresponded to individual song learning outcomes. We binned subjects into three learning categories: (i) ‘High-accuracy copiers’, who sang at least one identifiable, complex copy featuring 3 or 4 notes per syllable that were clearly attributable to a specific tutor model (SPCC values for the best-copied syllable = 0.79 ± 0.05; n = 4 birds); (ii) ‘Moderate copiers’, who sang at least one identifiable copy with 2 notes per syllable (SPCC values for the best-copied syllable = 0.74 ± 0.04; n = 5 birds); and (iii) ‘Low-accuracy copiers’, who sang single-note trills that could not be attributed to a specific tutor model (n = 4 birds). We found sharp cells in birds from all three categories, and their prevalence was statistically indistinguishable across the three learning groups (Fig. 3 red bars; Friedman chi-squared = 1.5, df = 2, p-value = 0.4724, Mann Whitney U value is greater than the critical value for all pair-wise comparisons indicating p > 0.05). This finding suggests that low-accuracy copying did not emerge from deficits in either the encoding of tutor songs in early development or in the persistence of tutor representations into adulthood. By contrast, bridge cells were significantly more common in high- than in low-accuracy copiers (Fig. 3 purple bars; Mann Whitney U_13.23_, df = 6, U value is less than critical indicating p < 0.05), with moderate copiers showing an intermediate number of bridge cells. We encountered a bridge cell in only one male in the low-accuracy group. We sampled similar numbers of single units from each learning category: 25 units from the four high-accuracy learners, 38 units from the five moderate learners, and 28 units from the four low-accuracy learners. Thus, while sharp cells were prevalent in birds from each group, bridge cells were more common in birds that achieved better learning outcomes. These data suggest that a greater prevalence of bridge cells is associated with higher-quality learning accuracy. The presence of bridge cells may therefore be a necessary precursor of learning accuracy or an outcome of accurate learning, but is clearly a defining feature of birds with better learning abilities.

In two additional tests we asked whether, across birds, bridge cell selectivity was specifically associated with imitative accuracy. First, we assigned ranks for the five tutor songs that were presented to each bridge cell based on (i) the electrophysiological responses evoked by those tutor songs (TSI, ranks of 1–5; 1 = highest value, 5 = lowest) and (ii) the corresponding pairwise similarities (SPCC) between those tutor songs and BOS. For 11 of 12 bridge cells, the tutor song for which the cell was most selective was also the best match for the BOS in terms of copying accuracy (Monte Carlo simulation for the average combined rank was significant at the α = 0.01 level; mean ± SEM of ranks = 1.08 ± 0.08; n = 12 cells from 8 birds). The likelihood this result would emerge by chance is further diminished when considering that swamp sparrows sing multiple song types (typically 2–5 types^24^) in their repertoires.

Second, we tested the relationship between two quantitatively-varying parameters: the copying accuracy of the BOS relative to each of the 5 tutor songs presented (SPCC), and the response strength (Z-score) of the bridge cell when presented with the copied versus non-copied tutor songs. For all bridge cells that we sampled, we observed a positive relationship (Fig. 3b,c; all slopes positive; F = 5.37, p < 0.0001). As in the rank-based analysis above, the tutor song that was the best acoustic match to the BOS (and thus the inferred model) also achieved the highest neural response score (Z-score value) in 11 of 12 cases. Thus, within each individual bird, the properties of bridge cell auditory response across tutor songs scaled directly with that individual’s best match to the copied tutor song. In other words, the neurophysiological response and acoustic analyses agree on the best match of BOS to tutor model for bridge cells. While the response properties of bridge cells identify the copied tutor song out of the set of tutor songs, the response properties do not predict the degree of accuracy by which the bird’s song matched the tutor or vice versa (Fig. S1).

### Segregation of cell types based on spike waveform

**Figure 4:**
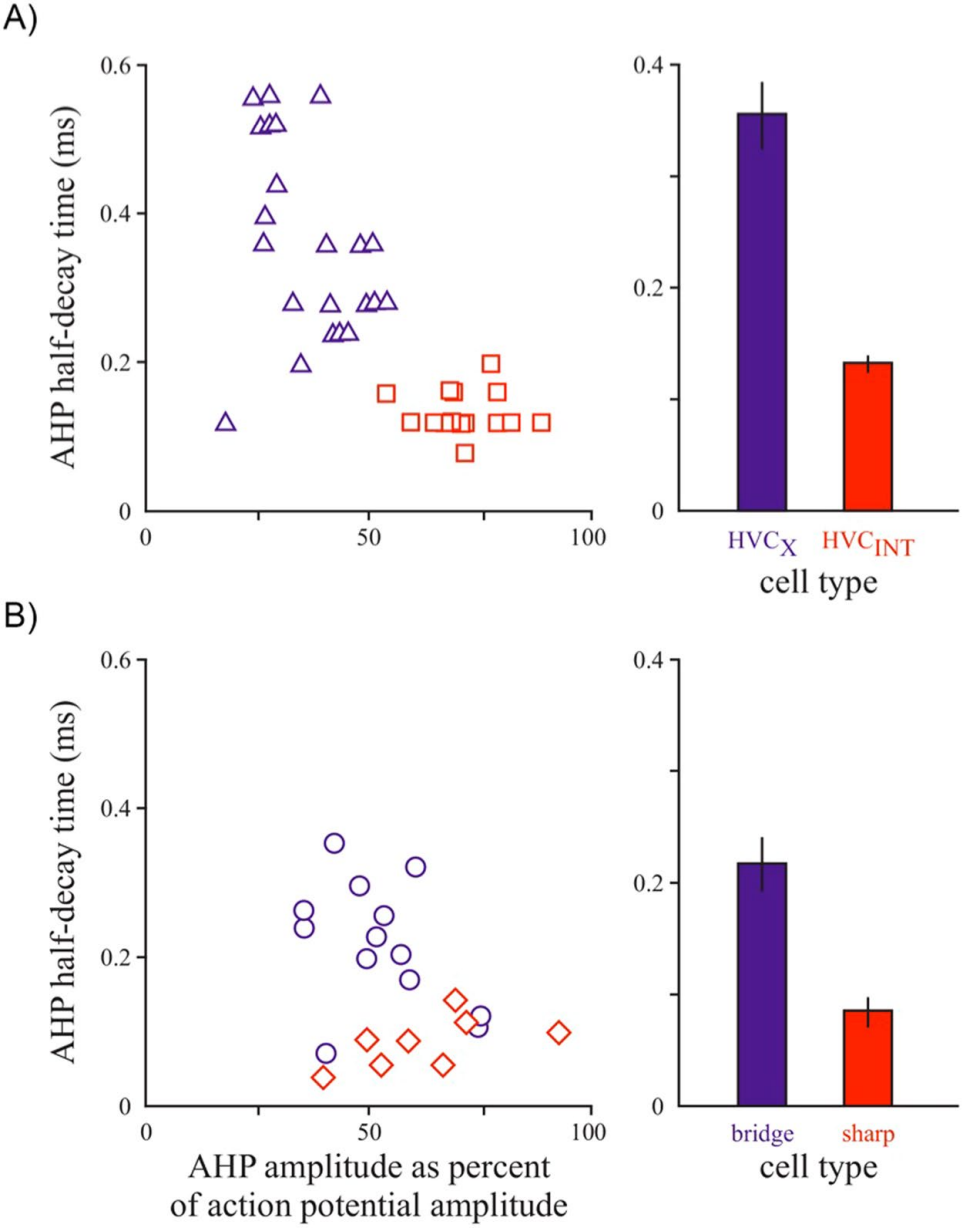
Action potentials for a separate group of awake-singing sparrows (n = 5 males) exhibit segregation of projection neurons to the striatum (HCV_X_ n = 23 units) versus local interneurons (HVC_INT_ = 19 units) based on waveform (**A**) and a similar segregation for bridge versus sharp cells in HVC recorded from learners in this study (**B**). Left panel (**A**) shows the relationship for AHP half-decay time vs. AHP amplitude as percent of action potential amplitude for antidromically identified HVC_X_ cells (purple triangles) vs. HCV_INT_ cells (red squares); right panel shows summary of the same data for AHP half-decay time only (p < 0.001). (**B**) Action potentials for the 13 males in our song-learning study show similar results for bridge cells (purple; n = 12) and sharp cells (n = 8; red); AHP half-decay times also significantly differ, p < 0.01.

To gain further insight into the identities of sharp vs. bridge cells, we quantified features of their action potential waveforms. Sharp cells and bridge cells differed significantly in the time required for extracellular voltage to return to baseline following the peak after-hyperpolarization (AHP half-decay; Fig. 4; Wilcoxon RS test, p < 0.01, n = 20 cells from 13 birds). Sharp cells returned to baseline significantly faster, expressing on average only 38% of the AHP half-decay time observed in bridge cells. This difference matches the distinction between HVC interneurons (HVC_INT_) and neurons that project to the avian striatum (HVC_X_ cells, Fig. S3).

A separate analysis of HVC spike waveforms sampled in awake and freely behaving swamp sparrows revealed that HVC_INT_ and HVC_X_ cells also differed significantly in AHP half-decay (Fig. 4; Wilcoxon RS test, p < 0.001, n = 23 HVC_X_ cells and 19 HVC_INT_ from 4 birds), with HVC_INT_ expressing only 37% of the AHP half-decay time observed in HVC_X_ cells (Fig. S3). Together, these data support the putative identification of bridge cells as HVC projection neurons and sharp cells as interneurons. These two cell classes differ in their sensitivity to real-time auditory feedback^25,26^, which could thus facilitate different aspects of model-copy error correction. Confirming the anatomical identity of HVC sharp versus bridge cells will be an important goal of future studies.

### Neural response structure and peak versus consistent spiking

Swamp sparrows differ from other songbird species like white-crowned sparrows^27^ and zebra finches in that swamp sparrow song consists of a single syllable repeated in a trill. This song syntax affords the technical advantage that any cell tuned to a specific note or element in the song responds to that element many times throughout the two-second stimulus during cellular recordings. For the majority of bridge neurons, cells showed consistent sustained spiking throughout the stimulus (see Fig. 2 BOS copied, Fig. S2). However we observed some momentary peaks in spiking in response to some stimuli, such as BOS (e.g. Fig. 2 BOS2). To address the degree of a short peak in spiking versus a sustained, consistent spiking by cells to tutor and BOS stimuli, we binned spike action potentials into 100ms sliding windows and determined the absolute maximum sum of spikes/100ms. The maximum sum of spiking in these bins further bolstered the idea that our categorized “bridge” neurons were not BOS-only selective, but were dually selective and showed similar spiking for the copied tutor song as for BOS (28.1 ± 3.5 sum spikes per 100 ms bin to BOS-copied, compared to 22.5 ± 4.9 to tutor-copied).

In over one-third of the sampled bridge cells, both the selectivity (TSI) and maximum spiking were higher in response to the copied tutor song than to BOS (i.e. higher tutor selectivity). For all the remaining cells, the maximum per rolling bin of 100ms was similar for TUT and BOS, except for one case. For this one case, the cell met our criteria for inclusion by TSI, and the maximum spiking during the tutor song was 157% of the other non-copied BOS. Lastly, for two males’ cells, a different BOS (not copied) in the males’ repertoire also showed high peak spiking, but for these cells the peak spiking was restricted early onset peaks rather than sustained spiking throughout the two-second trilled stimulus as was seen in the copied tutor and BOS-copied stimuli. For these two males’ cells, the onset spiking occurred precisely during the presentation of the introductory notes (exemplified in Fig. 2 BOS2), but was not sustained during the trilled (learned) portion of the song.

It is not surprising that cells in the swamp sparrow HVC show some recognition for another BOS in the males’ repertoire, yet these responses were different than the sustained magnitude of those to the copied BOS and selected tutor song. Generally, for all cases of the selected tutor and BOS-copied, we found that the highest bin values and absolute maxima of spiking values occurred throughout the trilled portion of the swamp sparrow song. Thus, analyses of both the whole-song TSI and maximum spiking return the same results for the trilled portion of learned swamp sparrow song, revealing that these cells are maximally responsive and selective for two stimuli – the tutor model and the birds’ copy of that model.

## Conclusions

Our findings are consistent with the hypothesis that the selective activity of bridge cells integrates song model memorization and imitative accuracy. These bridge cells have never before been seen in adult birds, nor in the HVC, and have not previously been tested in their selectivity for one out of multiple possible tutor songs. Prior studies have demonstrated that juvenile songbirds possess neurons with dual representations of songs, including tutor and autogenous songs^2,14,28–31^. Notably, Solis and Doupe^14,32^ reported cells dually selective for BOS and tutor songs within the LMAN of juvenile finches, and their work suggests bridge cells may be present in juveniles as well. Recently published work by Yanagihara and Yazaki-Sugiyama, also on juvenile zebra finches, showed similar results in the NCM, in which birds with significant tutoring experiences exhibit cells that are dually responsive to BOS and tutor songs^33^.

Our results build on these studies in three ways. First, while dual representation of model and copy were described previously in the juvenile song-learning period, we here report such a connection in adult birds past song crystallization. Second, while zebra finches sing a single song type copied from one tutor, swamp sparrows are a repertoire species possessing multiple BOSs memorized from multiple tutors, which therefore substantially reduces the likelihood of a cell showing strong selectivity for both a certain song type in its own repertoire and the corresponding tutor song (of many presented), especially for the tutor from which the BOS was copied. Here we further define the dual selectivity of bridge cells for song model and copy. Third, and perhaps most importantly, we identified a quantitative relationship between variation in learning accuracy and variation in single unit responses.

Our work expands on prior findings from zebra finches by identifying individual HVC bridge cells whose response properties are related both to specific models learned during juvenile development and to their matched sensorimotor representations in adulthood, against a backdrop of song repertoire learning and production. The prevalence and response properties of bridge cells are as likely to be an outcome of accurate song learning as a mechanism for it. Bridge cells may therefore function as an active link between current songs in birds’ repertoires to the tutor models of a specific song type. A connection between tutor models maintained in adulthood could serve for song matching or repertoire matching in species like swamp sparrows that use matching in aggressive interactions.

We have shown that the response properties of bridge cells in each bird reflect variation in individual learning accuracy. Thus, imitative learning ability scales not only with neural attributes like dendritic arborization and myelination^34–39^, but also with successful bridging of internal models and their eventual copies within individual sensorimotor neurons. The connections between tutor model “templates” and learned renditions of those models may differ between species for which the sensory and sensory motor phases do or do not overlap and as well as for birds that are either closed- or open-ended learners^40^, the latter of each group may require persistent representations of tutor models into adulthood that are later matched to birds’ renditions of those models. The potential importance of individual cells in these processes is supported in recent work by Vallentine *et al*.^41^, which showed that song is learned in sequential bits, and that each fixated bit is protected from further developmental plasticity by inhibition at the level of single neurons in HVC. Bridge neurons may be important for this process by selectively representing the specific song element that the bird is currently perfecting. More broadly, neurons with such properties likely play a central role in acquisition and or maintenance of learned behaviours. Moreover, because female sparrows show mating preferences for males that produce songs copied with high accuracy^42^, bridge cells also may be active targets of sexual selection.

## Methods

### Study animals and housing

All methods and experiments were performed in accordance with relevant guidelines, regulations and with the approval of the University of Massachusetts Institutional Animal Care and Use Committee (protocols #28–10–02 and #2010-0030). We collected swamp sparrow nestlings, four to eight days post-hatch, from nests in a population at the Quabbin Reservoir in Franklin County in western Massachusetts, USA, in June and July 2009. Birds were reared in sterilized swamp sparrow nests until fledging, then in groups of 5–8 in common cages (84 × 74 × 44 cm) to independence, and finally in individual cages (46 × 22 × 26 cm) throughout the length of the study. Birds were maintained at a naturally varying light:dark schedule, and had access to perches, semi-weekly baths, and *ad libitum* water. Nestlings and fledglings were fed a blended mixture of commercial turkey feed, carrots, eggs, ground beef, calcium derived from oyster shells, and vitamin powder. Birds were reared in two treatment groups (control, experimental) as part of a separate behavioural study assessing the effects of developmental stress on vocal performance^43,44^. Control nestlings and fledglings were fed until sated (until the cessation of begging behaviour), the volume of food consumed was measured to the nearest 0.05 mL^43–45^. Birds in the experimental treatment were fed 70% of the food volume their control age-related birds had accepted that hour. As birds grew to independence, their diet gradually transitioned to *ad libitum* seed but also included a dry food supplement and mealworms, given in a ratio by weight or number of 10 control: 7 experimental. Once birds achieved independence, they were housed in individual cages contained within walk-in acoustic isolation chambers for daily song training. We measured each bird’s mass to the nearest 0.1 g daily from age 4–60 dph and every other day from day 60–120 dph. Importantly, the variables of central interest to this study (wide variations in vocal imitative accuracy, the presence of sharp and bridge HVC cells) spanned the two treatment groups and were statistically indistinguishable in every parameter of interest, and so we made no further distinction between the treatment groups in our data analyses.

### Song training regimes

Training began at 9–29 days of age and continued until 118–148 days of age, thus encompassing the sensitive period for song memorization and acquisition in this species^46^. Tutor song models were constructed from ten swamp sparrow songs recorded previously from the Quabbin Reservoir population. Swamp sparrow songs are approximately two seconds in duration, and are comprised of a syllable repeated that contains between two and five notes each. To construct each training model, we clipped out a single syllable from each natural song, made digital copies of these syllables, and combined syllable copies in sequence to reconstruct songs at desired trill rates (e.g.^18^, and using Signal Software 4.1, Engineering Design 2003). Two control songs in each regime were reconstructed at the same trill rate as the original wild-recorded songs (“original trill rate” models), and the remaining eight songs in each regime were experimentally modified, via shortened inter-note intervals, to have higher trill rates while retaining their original syllable structure (“high performance” models). Wild-recorded songs used to construct training songs ranged in trill rate between 6.2–10.6 Hz. High performance models were increased to 115% to 155% of their natural trill rates, resulting in training model trill rates of 7.6–16.5 Hz, therefore four of these increased trill-rate songs still fell within the natural range of trill rates.*

Training songs were played at a rate of one song every 10 seconds, at a SPL of 80 dB at 1 m. This approximates what young birds experience in the wild. Each tutor model was played for six minutes, twice a day, and presented in random order. Song training was conducted during two 1-hour intervals each day – one hour was initiated within two hours of the lights turning on (roughly dawn) and the other hour was initiated within 4 hours of the lights turning off (roughly dusk).

From April through late June the following year (2010), we recorded the vocalizations of each bird for four hours in the morning, two days each week, as they sang in individual recording chambers. We recorded each bird on a weekly basis until birds had crystallized their song type repertoires. We recorded males again in 2011 when birds were 2 years old, to obtain additional recordings of each bird’s crystallized repertoire. For each copy by an individual bird, one rendition was chosen from each of the last five days of recording in 2011 for quantitative analysis of copying accuracy.

### Copying accuracy – Spectrographic cross-correlation analysis

We quantified the copying accuracy of each BOS (birds own song) from tutor models using spectrographic cross-correlation analysis (SPCC), a method which assesses the similarity between model songs and their copies in terms of time-varying frequency and amplitude structure (SIGNAL 4.0). SPCC values range from 0 to 1, with 1 indicating a perfect copy. Songs with at least two notes per syllable were assigned to corresponding tutor models by visual inspection of note structure and sequences within syllables from spectrograms (DLM and JP independently and by spectrographic cross correlation (SPCC; as in refs^11,18^). Cross-correlation scores generally match designations of song similarity based on visual examination of spectrograms^15,18^. For songs for which a tutor model could not be readily identified, best-matches to putative tutor songs were determined by SPCC. We generated SPCC copying accuracy values for both whole syllables and individual notes within the syllable for each copy. SPCC calculations were performed on spectrograms constructed with 128-point fast Fourier transformations (FFTs), 100 time steps, and within a frequency range of 1.5 to 10.5 kHz.

Analysis of the 33 distinct song types crystallized by males in this study showed that some song types may not have been copied at all, showing structural deficits (e.g., single-note syllables with atypical phonological structure) that parallel deficits in songs of swamp sparrows reared in isolation^47^. For song copies that included multiple notes per syllable (all song types from moderate to high-accuracy learners), the copying accuracy of a BOS to any of the ten potential models averaged 0.42 ± 0.04 syllable and 0.50 ± 0.15 for notes, while the values for any BOS to its best match tutor model were 0.76 ± 0.11 (n = 18 syllables) and 0.75 ± 0.09 (n = 55 notes). BOSs composed of only single-note per syllable trills were unassigned to a best match. For these single-note trills, notes were compared to tutor notes, but a clear assignment was not made because of the similarity of note categories in swamp sparrow song structure.

### Electrophysiology

Electrophysiological recordings were conducted on males 730–790 days of age. Bilateral craniotomy to permit access to HVC was performed under 20% urethane anesthesia (4 × 30 µl intramuscular injections over a 3–4 hr period) and following 2% lidocaine application to the scalp. HVC was located at 2.4 mm lateral from the bifurcation of the mid-sagittal sinus. A stainless steel head post was attached to the rostral skull via dental acrylic/cyanoacrylate. After craniotomy, the dura mater was carefully resected within the boundaries of the craniotomy over HVC. The bird was stabilized on a head-post anchor stage on an air table (TMC) inside a sound-attenuation booth (Industrial Acoustics), and kept warm with a custom body wrap and a direct current heating pad (FHC Neurocraft). Extracellular HVC recordings were carried out similar to established methods^22,48^. Briefly, a carbon fiber extracellular electrode (0.5–1.2 MΩ; Kation Scientific) was advanced into HVC using a hydraulic micromanipulator (Narishige) in both hemispheres. Search stimuli included the bird’s own song repertoire (BOS), conspecific song and white noise. HVC sites were located based on characteristic vigorous multiunit activation in response to BOS playback (whenever possible sites were verified within HVC following sacrifice and perfusion; 40 µm histological sections). We note that HVC responses in anesthetized sparrows are less temporally-precise than in the awake state, but maintain high selectivity for preferred stimuli regardless of behavioural state^21,22,49^.

Experimental sound stimuli included the birds’ own songs (BOS’s), a novel conspecific song (CON; recorded from the local population of swamp sparrows, but not used in tutoring), and at least 5 separate candidate tutor songs from the library of ten tutor songs presented to each male during development. Due to logistical constraints, see also^22^, not all tutor song stimuli could be tested in each recording (5 of 10 tutor songs per recording). However, each HVC site received all 10 tutor song stimuli in two successive recordings. All stimuli were bandpass filtered (0.5–10 kHz; Adobe Audition) and presented 15 times in randomized, interdigitated order at an interstimulus interval of 10 ± 2 sec at ~ 70 dB SPL. Recordings were amplified (10,000x), bandpass filtered (0.3–5 kHz; A-M Systems 1700) digitized at 20 kHz (Micro 1401; Cambridge Electronic Design), and stored on a computer using Spike 2 software (CED).

Single units were identified using Spike 2 sorting algorithms, via a combination of waveform template characteristics and principal component analyses, as described previously^50,51^. Only units meeting refractory period criteria were including in the analysis (the interspike interval for each cell within 1 ms < 1.0% of all ISIs). Peristimulus time histograms (10 ms bin size) were used to evaluate the auditory response properties of each cell, and paired t-tests were used to determine whether the auditory-evoked activity for each cell differed significantly from its baseline activity (activity during stimulus presentation compared against the activity of the same cell during an equivalent amount of time immediately preceding the stimulus onset; “response strength” as quantified in^14,20^). To standardize comparisons of response strengths across playback stimuli, cells and animals, auditory responses were z-transformed (expressed as Z-scores) for each cell as in prior studies^22,48^. Using these methods, we isolated between 1–16 single units per male (mean ± SEM = 7 ± 1.4), and these units distributed evenly across learning groups (low (n = 4 birds) = 28 units, moderate (n = 5 birds) = 38 units, high (n = 4 birds) = 25 units).

We then evaluated the selectivity of all the presented stimuli using first d’ (as described above^14,20^) to the conspecific novel songs and to TSI (as described above). We argue that TSI is a conservative measure for selectivity, as based on prior literature, one would expect neural responses to be higher in response to any tutor song than to a novel conspecific song, to a reverse song, or to a heterospecific song, thus when d’ is set to the tutor song generating the second-highest response, a cell would have to be highly selective to have a high TSI.

Using both d’CON and TSI, we were able to determine if cells were selective for various stimuli including BOS, a single tutor song, or were dually selective. We examined all singly, dually, and multi-selective cells out of the entire population of roughly 90 single units. We report every cell which met our TSI (d’(second highest tutor) criteria (Table 1) that was dually selective for BOS and any other stimulus. No cells were dually selective for BOS and novel conspecific songs. Some cells generally responded to two or more BOSs (BOS-selective) or two or more tutor songs (which we categorized as tutor responsive, but not tutor selective).

### Chronic electrophysiological recordings in awake subjects

Chronic recordings were performed with the approval of the Duke University Institutional Animal Care and Use Committee (protocol A023-03-01) compliant with state and federal regulations governing the capture and use of wild birds. Some of the cells for which spike properties are described here were also characterized according to their auditory response properties in previous publications, but the spike properties described here were not previously reported^22,52^. Briefly, individual HVC neurons were identified in awake and freely behaving adult male swamp sparrows using antidromic stimulation and were recorded using a microdrive system as described previously^22,50^. Individual units were identified using antidromic stimulation via bipolar electrodes placed in Area X and RA (n = 5 males, HCV_X_ n = 23 units, HVC_INT_ = 19 units) (40). In antidromic identification, HVC_X_ units displayed fixed-latency action potential responses to stimulation in Area X but no response to stimulation in RA^22,52^. In contrast, HVC_RA_ units (not described in this study but described here for the sake of clarity) displayed fixed-latency action potential responses to stimulation in RA but not in Area × ^22,52^. Each of these classes of projection neuron could be distinguished from HVC interneurons, which expressed variable-latency responses to stimulation in either RA (see also^53^) or Area X and occasionally to stimulation at both sites.

### Statistical Analyses

To investigate whether high-accuracy learners exhibited a greater number of sharp or bridge cells than did low-accuracy learners, we tested the number of units for each individual per group (Mann-Whitney U tests). Using a Monte Carlo analysis, we calculated the likelihood that, for any unit, a tutor song would achieve the highest neural response (Z score) and that the same tutor song would also be the best match to the BOS. We ranked the five tutor songs presented to each unit (ranked 1 through 5 with 1 reflecting the best performance) by both Z-score (one ranking of 1–5) and SPCC value comparing BOS to the tutor song (another ranking of 1–5). Bridge cells by definition achieved the highest Z score, and received ranks of 1, therefore we averaged the ranks for the copying accuracy across males after nesting units within individual. With the Monte-Carlo simulation, we created a distribution likelihood from a simulation of 1 million possible combinations, which generated confidence intervals and alphas against which to test the average copying accuracy ranks. The critical values for distribution of ranks under null with a mean rank of 3, were as follows: at the 1% level: (less than 1.7500, greater than 4.2500), at 5% level: ( < 2.00, > 4.00). We then tested the observed averages against those critical values.

In regression analysis comparing the neural responsiveness to copying accuracy, we plotted the Z score for each tutor song presented to the unit (an average neural response to stimuli) against the copying accuracy comparing the BOS to each tutor song presented. We then tested whether the slopes for each bridge cell differed from zero. Again we nested unit within individual and performed this analysis once using all five tutor songs presented (a comparison of all tutor songs individually) and again using only the top tutor song for which the unit was most selective and the average of the other four tutor songs (a categorical comparison of the top model versus all other models).

### Ethical Statement

All methods and experiments were performed in accordance with relevant guidelines, regulations, and approval of the University of Massachusetts Institutional Animal Care and Use Committee (IACUC) (protocols #28-10-02 and #2010-0030) and approval of the Duke University IACUC (protocol A023-03-01) and are compliant with state and federal regulations governing the capture and use of wild birds.

### Data Availability

The datasets generated during and/or analysed during the current study are available from the corresponding authors on request.

## Electronic supplementary material

Supplementary Information
