# Supplementary material for: A neuronal signature of accurate imitative learning in wild-caught songbirds (swamp sparrows, Melospiza georgiana)
Source: Sci Rep. 2017 Dec 11;7:17320. doi: 10.1038/s41598-017-17401-2 (PMC5725582; doi:10.1038/s41598-017-17401-2)
Supplement: Supplementary file 1 — Supplementary Information [file 41598_2017_17401_MOESM1_ESM.doc]

**Title: A neuronal signature of accurate imitative learning in wild-caught songbirds (swamp sparrows, *Melospiza georgiana*)**

**Supplemental Information**

**Authors:** Moseley, Dana L.^1,2,3*^, Joshi, Narendra R.^4^, Prather, Jonathan F.^5^, Podos, Jeffrey^1^, and Remage-Healey, Luke^4*^

**Affiliations:**
^1^Department of Biology, University of Massachusetts, Amherst, MA 01003, USA

^2^Smithsonian Institution, Migratory Bird Center, National Zoological Park, PO Box 37012 MRC 5503, Washington, DC 20013-7012

^3^Department of Biology, James Madison University, Harrisonburg, VA 22807

^4^Department of Psychological and Brain Sciences, University of Massachusetts, Amherst, MA 01003, USA

^5^Department of Zoology and Physiology, Program in Neuroscience, University of Wyoming, Laramie, WY 82071, USA

*Correspondence to: [MoseleyD@si.edu](mailto:dlmoseley@wm.edu) or healey@cns.umass.edu

**Supplemental Information**

**Figure S1**

Across the population of HVC bridge units, the relationship between TSI (a) was not significantly correlated with copying accuracy (the degree to which the BOS matched that tutor song as measured by SPCC, R^2^<0.03, p = 0.602, Pearson’s correlation coefficient = 0.168). b) Also, d’BOS was not correlated to copying accuracy (R^2^ = 0.183, p = 0.167, Pearson’s correlation coefficient = 0.426). For three of the units, the response to the top tutor was higher than to BOS, while for the rest of the units the response was lower than to BOS, however all units met the criteria listed in Table 1 – the response to the top tutor as compared to BOS was never more than twice as large nor less than half as small as measured by TSI and d’CON. These results indicate the degree of similarity between tutor model and copy was not predictive of the strength of neural selectivity.

**Figure S1**

a.


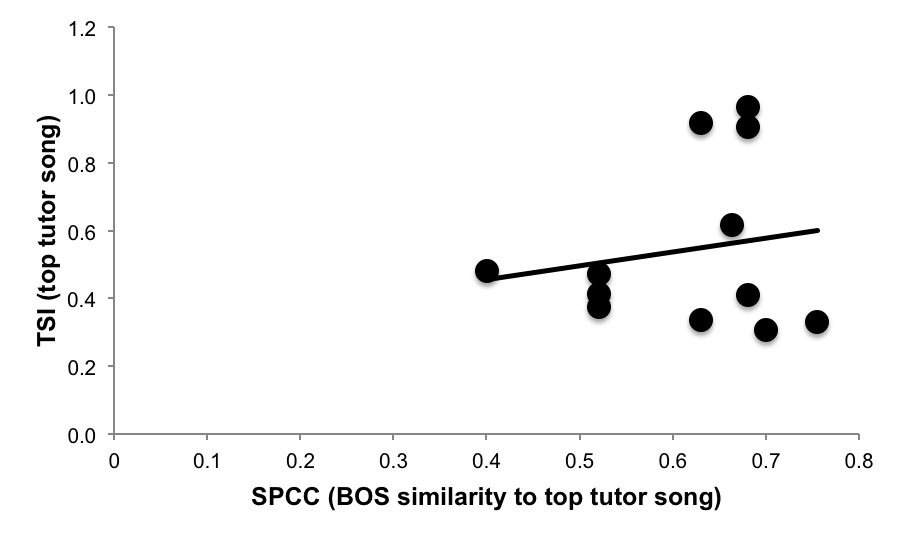


b.


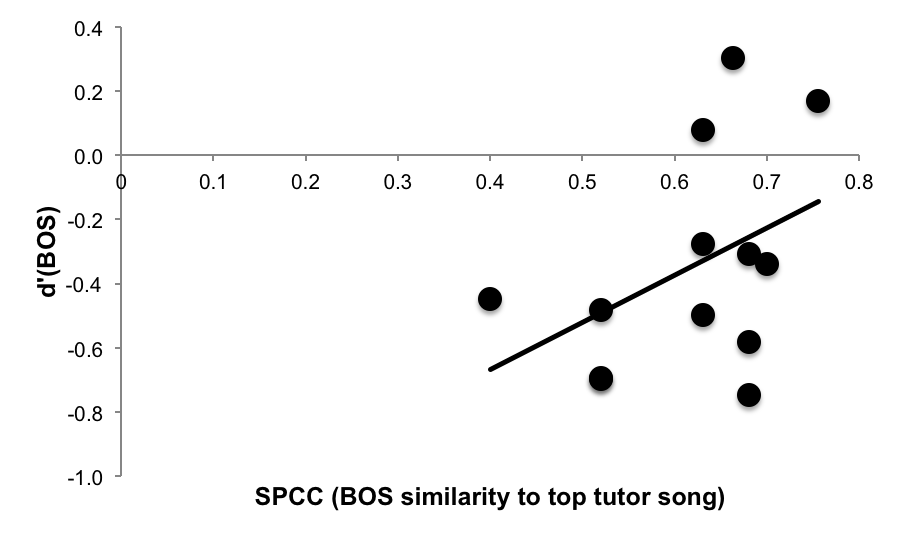


**Figure S2**

Raster plots (top), peristimulus histograms (middle), and spectrograms (bottom) are depicted for bridge cells responding to top tutor stimuli from four individuals that varied in their copying accuracy, spectrograms of the selected tutor stimulus and the corresponding BOS are presented to the right of each graph.

a) Male 12 reproduced tutor 8 with high copying accuracy, see Fig. 2 in the main document for additional information. b) Male 8 copied tutor 7 with moderate accuracy, c) Male 13 copied tutor 9 with moderate accuracy as well, but the reproduction was poorer than Male 8, and d) Male 7 was the one case in which a bridge cell was not selective for the best-match tutor model, as he produced a one-note trill which was matched by note using SPCC to a different tutor song, resulting in Tutor 4 as the second best note-match.

**Figure S2**

**Figure S3 -** Schematic of afterhyperpolarization half decay

The schematic illustrates measurements of single units in the HVC for their spike width, spike amplitude, peak voltage after-hyperpolarization, and time and voltage to return to baseline following the peak after-hyperpolarization (AHP half-decay). We quantified these features for swamp sparrows (*Melospiza georgiana*), N=13 males under anesthesia and N=4 awake and freely behaving males. Illustrated here, HVC interneurons (HVC_INT_) and neurons that project to the avian striatum (HVC_PROJ_ cells, Fig. S3) differ significantly in their AHP half-decay (Fig. 4; Wilcoxon RS test, p < 0.001, n = 23 HVC_PROJ_ cells and n=19 HVC_INT_ from 4 birds), with HVC_INT_ expressing only 37% of the AHP half-decay time observed in HVC_PROJ_ cells (Fig. S3). Bridge and sharp cells similary differed. Sharp cells returned to baseline significantly faster, expressing on average only 38% of the AHP half-decay time observed in bridge cells (AHP half-decay; Fig. 4; Wilcoxon RS test, p < 0.01, n = 8 sharp, n = 12 bridge cells from 13 birds).

**Figure S3**
